# Supplementary material for: The interactome of intact mitochondria by cross-linking mass spectrometry provides evidence for coexisting respiratory supercomplexes
Source: Mol Cell Proteomics. 2017 Dec 8;17(2):216–32. doi: 10.1074/mcp.RA117.000470 (PMC5795388; doi:10.1074/mcp.RA117.000470)
Supplement: Supplemental Data [file supp_RA117.000470_133922_0_supp_23978_fzffwf.zip › spectra_annotation/mito_spectra_annotation/372-1-7-1-17-1.pdf]

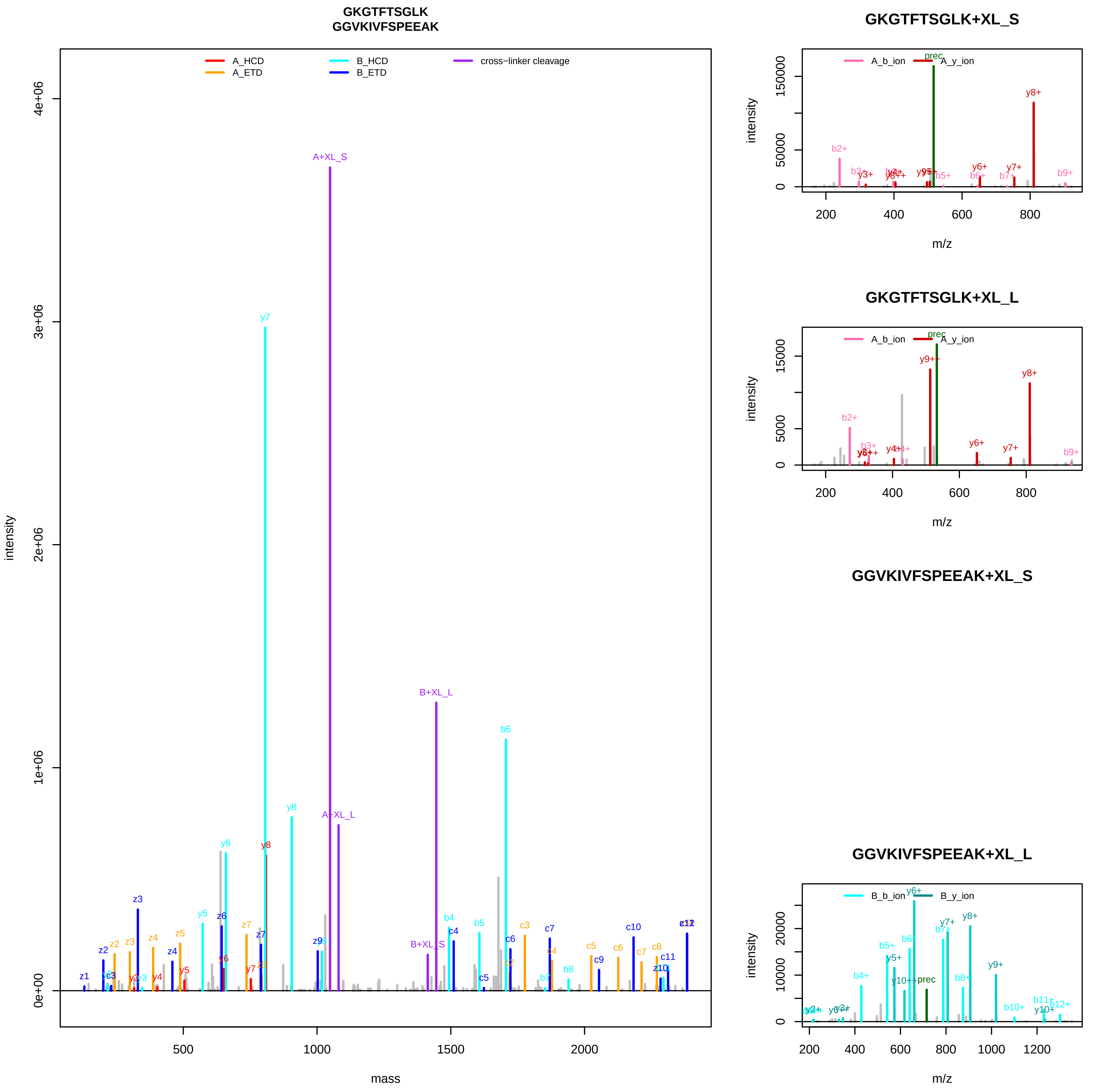
GKGTFTSGLK
GGVKIVFSPEEAK
A_HCD
A_ETD
B_HCD
B_ETD
cross−linker cleavage
intensity
mass
0e+00
1e+06
2e+06
3e+06
4e+06
500
1000
1500
2000
A+XL_S
y7
B+XL_L
b6
y8
A+XL_L
y6
GKGTFTSGLK+XL_S
A_b_ion
A_y_ion
prec
y8+
b2+
intensity
m/z
GKGTFTSGLK+XL_L
y9++
y8+
b2+
GGVKIVFSPEEAK+XL_S
GGVKIVFSPEEAK+XL_L
B_b_ion
B_y_ion
y6+
y7+
y8+
b5+
b6+
y9+
prec
